# Acupuncture to Promote Recovery of Disorder of Consciousness after Traumatic Brain Injury: A Systematic Review and Meta-Analysis

**DOI:** 10.1155/2019/5190515

**Published:** 2019-03-19

**Authors:** Li Tan, Liling Zeng, Ning Wang, Meijun Deng, Yinshi Chen, Tianyi Ma, Luhan Zhang, Zhenhua Xu

**Affiliations:** ^1^Guangzhou University of Chinese Medicine, Airport Road, Baiyun District, Guangdong, Guangzhou 510405, China; ^2^Guangdong Provincial Hospital of Chinese Medicine, Yide Road, Yuexiu District, Guangdong, Guangzhou 510006, China; ^3^The Seventh Affiliated Hospital, Zhongshan University, ShenZhen 518107, China

## Abstract

Traumatic brain injury (TBI) has become an economic and social burden for patients and their families. While acupuncture is an effective tool for promoting recovery of disorder of consciousness (DOC) following TBI, there have been no comprehensive meta-analyses and/or systematic reviews addressing this topic. The present systematic review and meta-analysis aimed to assess the therapeutic efficacy of acupuncture for DOC after TBI. All randomized controlled trials (RCTs) incorporating acupuncture, or acupuncture combined with other interventions for DOC after TBI, were included and assessed by two independent investigators. Six outcome indicators were assessed: Glasgow Coma Scale (GCS); Glasgow Outcome Scale (GOS); mortality; efficacy rate; activities of daily living (ADL); and functional comprehensive assessment. Direct comparisons were performed using RevMan 5.3.0 software, with results presented as mean difference (MD) for continuous outcomes and relative risk (RR) for binary outcomes. A total of 3511 patients from 49 trials were included. Pooled analyses indicated that acupuncture may have a superior effect on GCS score (MD=2.03, 95% CI :1.92 2.43,* Z*=16.54, and* P*<0.00001); GOS score (RR=1.23, 95%CI: 1.18 1.35,* Z*=6.65, and* P*<0.00001); efficacy rate (RR=1.48, 95%CI: 1.40 1.56,* Z*=13.49, and* P*<0.00001); ADL (MD=9.20, 95% CI:8.19 10.21, Z=17.84, and* P*<0.00001); and mortality (RR=0.50, 95% CI:0.38 0.67, Z=4.70, and* P*<0.00001). The results demonstrated that the acupuncture group fared better than the control group in the treatment of DOC after TBI. However, studies were generally of poor quality, and publication bias favoring positive studies was obvious. Therefore, rigorous evaluation standards and well-designed studies are necessary in future studies.

## 1. Introduction

Traumatic brain injury (TBI) is a global public health problem and one of the major causes of death and disability [1]. According to a survey by the World Health Organization, TBI will surpass many other factors as a main cause of death and disability by the year 2020 [2]. According to statistics, more than 50 million individuals experience TBI each year, and the total number of new TBI cases has been reported to approach 3.5 million per year in the United States [3], with 30-40% of deaths related to the injury. The mortality rate of TBI in China was 12.99/100,000 in 2013 and 17.1/100,000 in the United States, with traffic accidents and falls as the main causes [4–6]. TBI has become an enormous economic burden to families and society as a whole. TBI-related costs were estimated to be as high as €33 billion in Europe in 2010, of which indirect costs accounted for 59% and direct costs accounted for 41% [7, 8]. In the United States, it has been reported that aggregate direct and indirect costs increased from USD$60.4 billion in 2000 to USD$221 billion in 2009 [9].

The rapid development of emergency medicine and intensive care technology in recent years has clearly improved the survival of individuals who sustain TBI [10]. However, regarding severe brain damage, patients also experienced varying degrees of disorders of consciousness (DOC) after TBI, which seriously affects an individual's ability to perform the activities of daily living (ADL) and reduces the quality of life [11–13]. The early recovery of consciousness is closely associated with recovery in other functional domains, and the duration of DOC is an important prognostic factor in patients who experience TBI [13–15].

The annual cost for treatment and rehabilitation of TBI patients is nearly $400 billion, which is associated with tremendous inconvenience and economic pressure on patients, their families, and society [16, 17]. Therefore, studies investigating the recovery of patients' consciousness after TBI are important in the field of neurologic rehabilitation and have a great economic and social impact. There are many important treatment approaches for recovering consciousness, including pharmacological interventions, surgery, rehabilitation, and alternative medicine treatments such as acupuncture. Acupuncture has been shown to be an effective method when applied to traumatic injuries. A systematic review by Ma concluded that the use of acupuncture to treat spinal cord injury may have a beneficial effect on neurological and functional recovery. [18] Another review suggested that acupuncture is efficacious in acute TBI [19]. In China, acupuncture has been used for centuries to promote the recovery of consciousness after coma or other DOCs by increasing the excitability of nerve cells [20–22] and the supply of oxygen and blood flow in the traumatized area of the brain, which may be a potential mechanism for its rousing effects [23–25].

Although many studies investigating acupuncture for treatment of DOC after TBI have been published, they have not been systematically reviewed. Thus, the aim of the present systematic literature review was to assess the rousing effect of acupuncture on DOC after TBI.

## 2. Methods

### 2.1. Protocol and Registration

We had published a protocol of this systematic review in the PROSPERO database (identification number: CRD42018091226).

### 2.2. Data Source and Search Strategy

A systematic search of the literature was conducted in PubMed, Cochrane Library, Chinese Biomedical Literature Database (CBM), VIP, WanFang Database, and Chinese National Knowledge Infrastructure (CNKI) databases for articles published up to February 28, 2018. The searching terms include “coma”, “disorder of consciousness”, “minimally consciousness state”, “vegetative state”, “locked-in state”, “traumatic brain injury”, “brain injury”, “head injury”, “acupuncture”, and “electro-acupuncture”. The search was limited to studies published in Chinese and English.

### 2.3. Inclusion Criteria

All trials in which the intervention involved acupuncture or electroacupuncture separately, or acupuncture combined with other interventions, such as hyperbaric oxygen (HPO), traditional Chinese medicine (TCM) rehabilitation, and electrical stimulation, were included. There was no distinction for acupoints, manipulation, stimulation intensity, or the course of treatment. It is available for basic treatment or combined with rehabilitation training in the control group. The trials' outcome indicators had to include at least one of the following: Glasgow Coma Scale (GCS) or Glasgow Outcome Scale (GOS).

### 2.4. Exclusion Criteria

Trials that used transcutaneous electrical nerve stimulation and/or manual acupressure and DOC results from nontraumatic diseases, such as cerebrovascular disease, tumor, or toxicosis, were excluded. In addition, trials without a nonacupuncture group were also excluded.

### 2.5. Data Extraction

Data were extracted independently by two authors (LT and LZ) using a specifically designed data extraction form. Disagreements were resolved by assistance from the third author (NW). Data extracted from each study were summarized in a data extraction table. Items noted included the following: first author and year of publication; type of intervention; sample size; sex ratios; years; TBI's degree, duration of treatment, and main outcome indicators in each trial. For studies in which outcomes were assessed at several different time points, the measurement after the final treatment was selected. Meanwhile, if the duration of treatment described as applying according to the condition of disease, we attributed it to an unclear duration of treatment.

### 2.6. Quality Assessment

Two authors (MJD and TIM) evaluated the quality of included trials. The risk of bias was assessed using the risk of bias tool from the Cochrane Handbook of Systematic Reviews of Interventions [26] according to the following criteria: random sequence generation allocation concealment; blinding of participants and personnel; blinding of outcome assessment; incomplete outcome data; and other biases and descriptions of the risk of bias.

### 2.7. Data Analysis

RevMan 5.3 software was used for data analysis and Stata version 13.0 (StataCorp LP, College Station, TX, USA) was used for sensitivity analysis. Different outcome indicators were classified and analyzed. Relative risk (RR) was evaluated for dichotomous outcomes, while mean difference (MD) was used for continuous outcomes with 95% confidence interval (CI). Because there was significant clinical heterogeneity among the trials (*P*<0.1), a random-effects model was used in the meta-analysis, and a fixed-effects model was used for those with low heterogeneity (*P*>0.1). Potential publication bias was assessed using funnel plots.

## 3. Results

**Figure 1:**
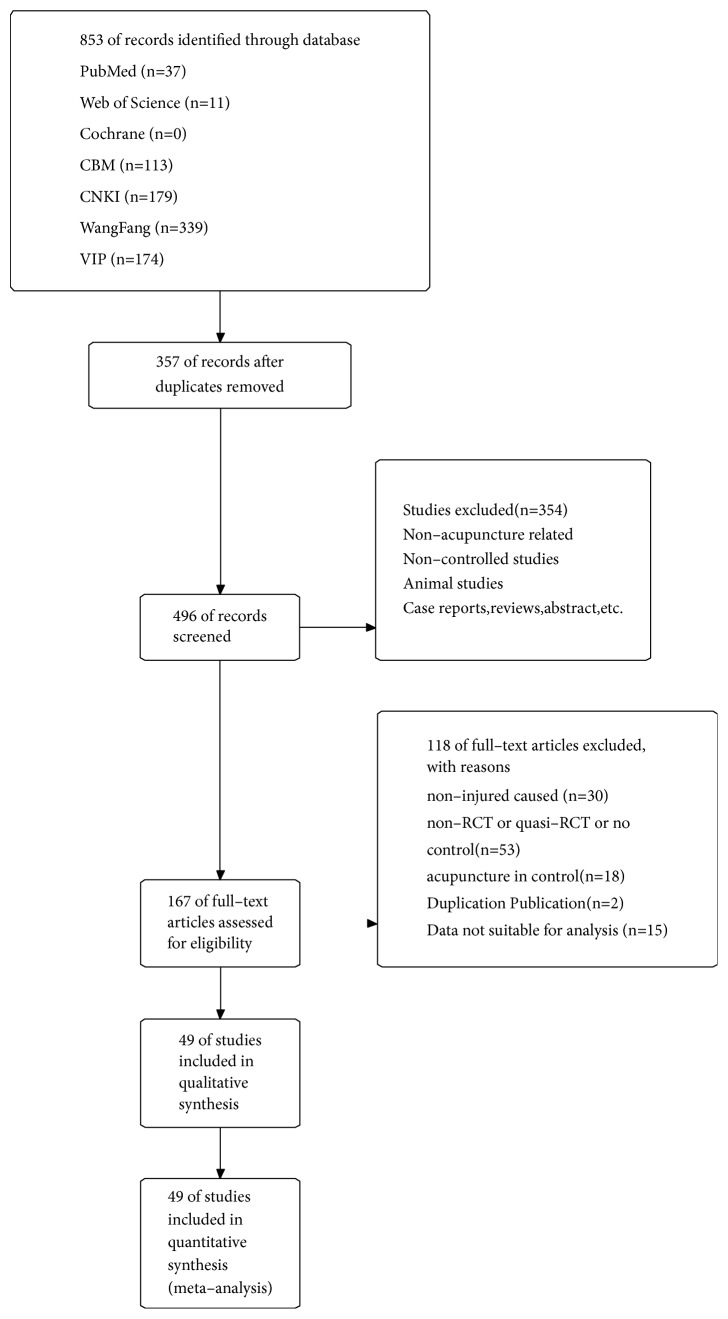
The screening flow diagram.

A flowchart depicting the search process and study selection is shown in Figure 1. Forty-nine studies including 3511 patients were ultimately included. There were 1800 participants in the acupuncture group and 1711 in the control group.

### 3.1. The Basic Characteristics

All trials were conducted in China and published in Chinese. The acupuncture and control groups were compared statistically according to sex, age, duration, and level of consciousness; baseline data were comparable. Regarding the intervention in the treatment groups, acupuncture was applied in 18 RCTs, electroacupuncture in 16, acupuncture combined with HPO in 6, acupuncture combined with TCM in 7, electroacupuncture combined TCM and HPO in 1, and acupuncture combined TCM and laser intravascular irradiation in 1. Thirty-seven studies reported GCS data before treatment, with sample sizes ranging from 29 to 126. Only 10 RCTs enrolled patients and reported the duration of TBI. The characteristics of the included trials are summarized in Table 1.

### 3.2. Quality Evaluation

**Figure 2:**
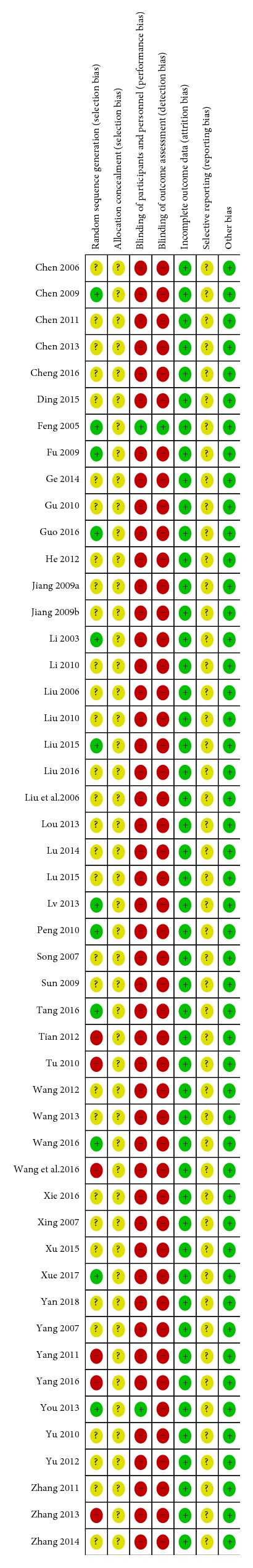
The bias of each study.

**Figure 3:**
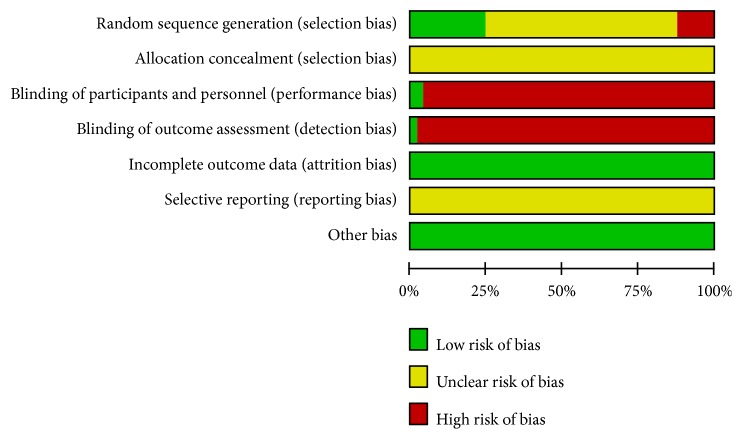
The summary of bias evaluation for the studies.

The majority of the included studies were assessed to be of generally poor methodological quality according to the Cochrane Handbook of Systematic Reviews of Interventions. Although “random allocation” was mentioned in all studies, only 12 RCTs reported the details of random sequence generation using a random numbers table. [21–23, 27–35]. There was no clear information regarding allocation concealment, and only 2 RCTs reported the blinding method [22, 32]. For incomplete outcome data, 1 study reported drop-out and exit of patients without any effect on the results, and 1 reported transfer from one hospital to another [29, 32]. Selective reporting was generally unclear due to inaccessibility of trial protocols. Only 7 trials reported funding support [25, 30, 32, 34–37]. The methodological quality of the included RCTs is shown in Figures 2 and 3.

### 3.3. Glasgow Coma Scale (GCS)

Thirty-seven trials reported the level of consciousness (according to GCS) before treatment [20–23, 25, 27–30, 32–58], and GCS score was used in 28 studies as the outcome measure [20–23, 25, 27–30, 32, 33, 35–39, 41, 42, 44–48, 54–56, 58]. Twenty-two studies used continuous data, and the statistic used was the difference in GCS score before and after treatment [20, 21, 23, 27–30, 32, 33, 36–39, 41, 42, 44–46, 48, 54, 55]. The GCS results showed high heterogeneity (*I*^2^=72%), so we did a subgroup analysis according to the treatment duration, TBI's degree (we define the GCS score between 3 and 8 as severe TBI, 8 and 12 as moderate TBI, and 13 and 15 as mild TBI), and intervention. From Table 2, results of subgroup of unclear treatment course [36, 42, 54] in treatment duration are the main source of heterogeneity (*I*^2^=96%) and show that there is no difference between acupuncture group and control (MD=1.34, 95%CI -0.24 2.92). Also, there were no differences between treatment group and control for results of subgroup of unclear degree of TBI patients in TBI's degree (MD=2.03, 95%CI: -0.06 4.12) and subgroup of acupuncture plus TCM in intervention (MD=0.83, 95%CI: -0.49 2.15). Test for overall effect demonstrates that the acupuncture could improve the GCS score (MD=2.03, 95%CI 1.54—2.52, Z=8.17, and* P*<0.00001) (Table 2).

### 3.4. Glasgow Outcome Scale (GOS)

**Figure 4:**
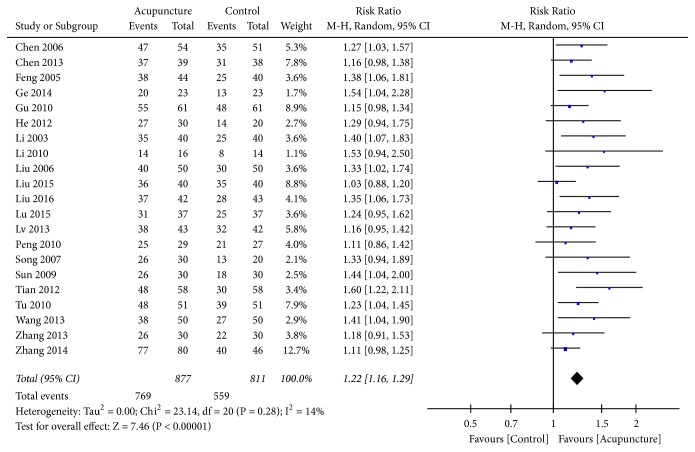
Forest plot of GOS.

The GOS was used in 28 trials, 25 of which used dichotomous data [22, 25, 31, 33–35, 40, 43, 45, 49–53, 57–66], and 2 were excluded because outcome times were unclear [50, 63]. Outcome was defined as good conscious state (GOS score ≥ 3/5) or poor (GOS score 1-2) as a simplified measure of level of consciousness outcome after TBI. The RR was selected to represent the count data. Low heterogeneity (*I*^2^ = 14%, P=0.28) was found after pooling the data; hence, a random-effects model was applied. The rectangle was on the right of the equivalence line, which indicated that acupuncture could improve the level of consciousness after TBI (RR=1.23, 95%CI 1.20 1.33], Z=8.88, and* P*<0.00001) (Figure 4).

**Figure 5:**
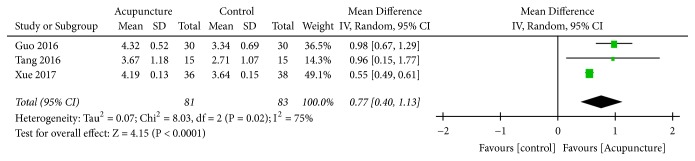
Forest plot of GOS.

Three trials [27, 29, 30] used continuous data with MD. The results indicated high heterogeneity (*I*^2^ = 75%,* P*=0.02); therefore, a random-effect model was applied. Pooled analysis revealed that acupuncture improved GOS score (MD= 0.77, 95% CI: 0.40 1.13,* Z*=4.15, and* P*=0.01) (Figure 5).

### 3.5. Mortality

**Figure 6:**
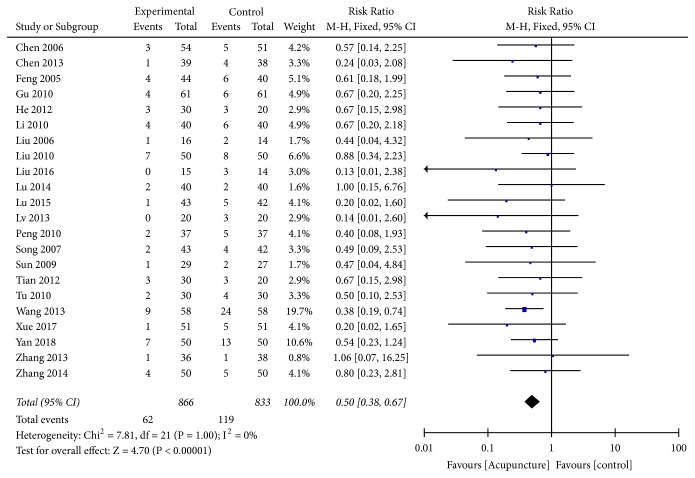
Forest plot of mortality.

Among the included studies, 24 [20, 22, 25, 27, 31, 33–35, 40, 43, 45, 49–53, 57–60, 62, 64–66] reported mortality data after acupuncture treatment. Due to low statistical heterogeneity (*I*^2^ = 0%,* P*=1.0), a fixed-effects model was applied, which revealed that mortality rates between the acupuncture and the control groups were significantly different (RR=0.50, 95%CI: 0.38 0.67,* Z*=4.70, and* P*<0.00001) (Figure 6).

### 3.6. Efficacy Rate

**Figure 7:**
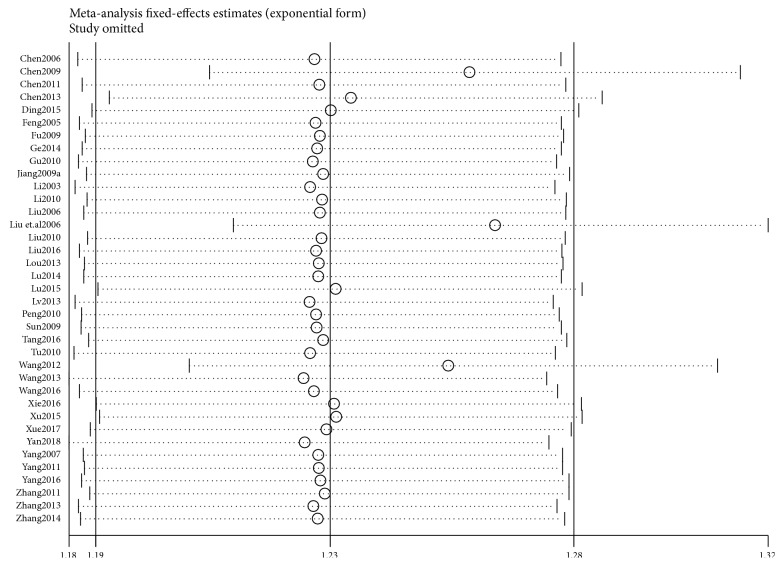
Sensitivity analysis.

**Figure 8:**
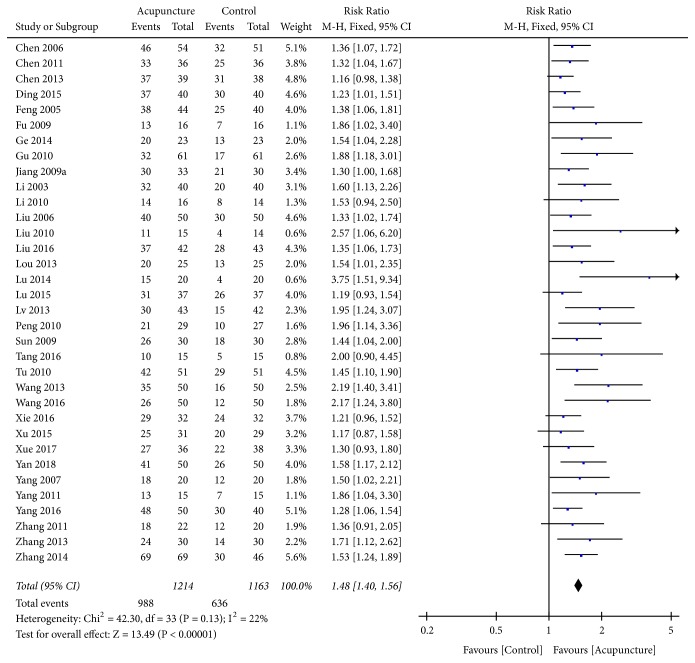
Forest plot of efficacy rate.

**Figure 9:**
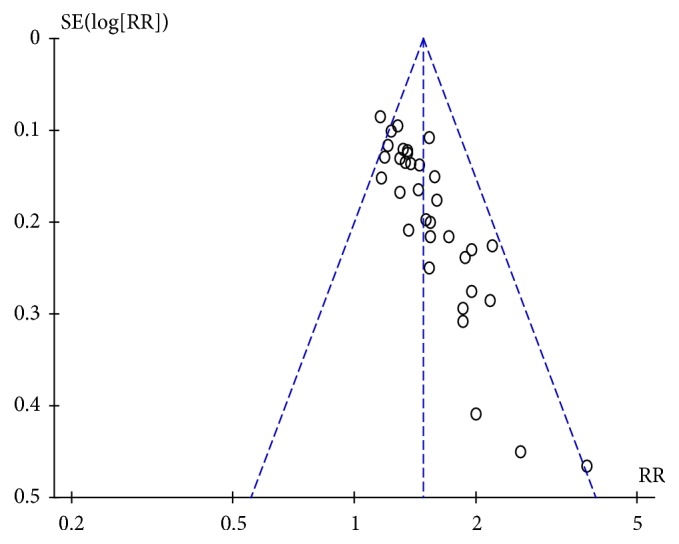
Funnel plot of efficacy rate.

In 37 trials [20–23, 25, 27–29, 33–35, 38, 40–44, 46, 47, 49–53, 55–64, 67–69], the clinical efficacy rate was used as the evaluation indicator with dichotomous data; therefore, RR was used to express the results. After combining the data, high heterogeneity (*I*^2^ = 69%,* P*<0.00001) was found; therefore, a sensitivity analysis was applied. The funnel plot revealed that 3 trials [21, 44, 56] deviated from the center line (Figure 7), which had significant influence on combined effect due to high sensitivity. The curative effect in the acupuncture group was found significantly higher than that of the control group. After high-sensitivity studies were removed, low heterogeneity was found after remerging (*I*^*2*^ = 22%,* P*=0.13); therefore, a fixed-effects model was used for analysis. The results revealed there was a statistically significant difference between the acupuncture and the control groups (RR=1.48, 95%CI: 1.40 1.56,* Z*=13.49, and* P*<0.00001) (Figure 8). The funnel plot demonstrated asymmetry, indicating publication bias (Figure 9).

### 3.7. ADL

**Figure 10:**
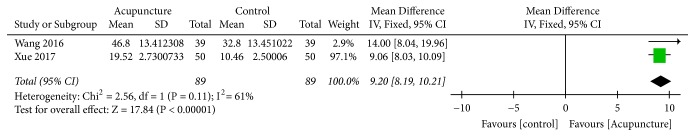
Forest plot of ADL.

Two trials [27, 39] used ADL to assess the curative effect and the continuous data were analyzed. Meta-analysis revealed that the acupuncture group demonstrated significantly improved ADL, with moderate heterogeneity (*I*^2^= 61%,* P*=0.11). The results indicated that improvement of ADL was 9.20 times higher than the control group (MD=9.20, 95% CI: 8.19 10.21, Z=17.84, and* P*<0.00001) (Figure 10).

### 3.8. Functional Comprehensive Assessment(FCA)

Among all included studies, FCA was used as an evaluation of neural function recovery in 2 investigations [23, 42]. Both demonstrated a better effect in the acupuncture group compared with the control group; however, the effect was not combined with high heterogeneity (*I*^2^ = 97%,* P*<0.00001).

## 4. Discussion

The results of the present review suggest that acupuncture may have a beneficial effect on the recovery of consciousness after TBI. According to these findings, acupuncture may improve consciousness levels evaluated using GCS or GOS scores, clinical efficacy, and lower mortality after TBI. Acupuncture may also help in rehabilitation. The mechanism for the beneficial effects of acupuncture in promoting arousal in TBI patients has been investigated in many animal experiments [70–72]. Cheng et al. [36] found that lowering the level of plasma D-dimer may be one of the therapeutic mechanisms of electroacupuncture in recovering consciousness.

However, these positive findings should be interpreted cautiously due to the high risk of bias in all of the included studies, the quality of which was poor overall. Among the 49 studies, the method of randomization was completely described in only 12 trials and incompletely described in 6, whereas the remainder merely only mentioned that “patients were randomized into two groups”. Without appropriate randomization, there is a strong possibility that some of these studies were not actually RCTs. Of all RCTs, only 2 reported blinding and none of allocation, which demonstrated exaggerated treatment effects and limited the reliability of the study results. In addition, only 2 trials reported dropouts and some trials did not report the missing data that led to the exaggerated treatment effects of acupuncture.

Despite the positive results, there were several obvious problems with the trials we analyzed. First, the sample size in many of the studies was too small to draw firm conclusions, and none of the included RCTs reported calculation of sample size. Second, treatment in the control groups could be a major source of bias, especially for clinical efficacy rate, which was subjective. All RCTs compared acupuncture versus basic treatment, and no sham acupuncture or placebo was used; moreover, the patients in these studies were not blinded as to whether they were receiving acupuncture or nonacupuncture. If participants had a preference for acupuncture relative to control treatment, it may have resulted in a greater placebo effect than basic treatment. Third, some trials used acupuncture combined with other interventions in the control group, which resulted in mixed effects and, in turn, made it difficult to evaluate the correlation between differences in acupuncture treatment and its therapeutic effectiveness. Thus, analysis of acupuncture alone or combined with other interventions in the control or experimental groups may increase reliability. Meanwhile, the duration and frequency of acupuncture, acupoint selection and stimulation parameters, and time of electroacupuncture varied among the RCTs, which may have contributed to the heterogeneity among the included trials. Fourth, the duration and grade of TBIs were different in the included trials and not reported in many RCTs, which may have led to different outcomes in clinical efficacy rate, GOS score, and mortality and was an additional source of heterogeneity in the meta-analysis. It has been established that high GCS scores are likely to result in good outcomes, and patients with DOC may regain consciousness more quickly. Fifth, the funnel plot demonstrated asymmetry, indicating potential publication bias against “negative” studies due to journal' preferences for positive research. Moreover, it is difficult to judge whether there were unreported negative research results given the inaccessibility of the study protocols. Adverse events and safety profiles of acupuncture were not reported.

## 5. Conclusion

To our knowledge, this was the first systematic review and meta-analysis to investigate acupuncture for DOC after TBI. Although the results suggest that acupuncture produced superior effects on the recovery of consciousness in the included trials, the above limitations make this effect questionable and difficult in drawing definitive conclusions. In the future, there is a need for more high-quality studies designed to investigate the rousing effect of acupuncture on patients with TBI. In particular, more effort and attention should be dedicated to improving the quality of studies to enhance internal validity. For example, inclusion criteria, sufficient sample size, truly randomized methods and adequate concealment of allocation, blinding of investigators and participants, and outcome assessors should be unified to minimize performance and assessment bias. Moreover, widely recognized and standardized outcome measurements, such as GCS and GOS, should be adopted to ensure reliability, comparability, and validity and to present results clearly in sufficient detail. Additionally, study protocols and clinical trial registration are an essential part of research. Researchers should be encouraged to devote special attention to monitoring and reporting adverse events regarding acupuncture intervention, quality of life, long-term efficacy, and objective outcomes (such as electroencephalography and functional magnetic resonance imaging). Even if acupuncture demonstrated an effect superior to control in our study, more evidence as to whether it has significant advantages over sham acupuncture at the same acupoints is needed.

## Figures and Tables

**Table 1:** The basic characteristic of the included studies.

Studies	Simple Size (T/C)(M/F)	Mean age (T/C)	Intervention (T/C)	GCS before treatment (T/C) (GCS Scores/number)	TBI's Degree	Duration of Treatment	Outcome measure
Fu 2009	16(12/4)/16(14/3)	41.8±18.5/36.8±18.5	EA/BT	4.06±1.06/3.87±1.02	S	30 D	GCS
He 2012	30/20	NR	EA/BT	NR	S	1 M	GOS
Yu 2010	34/34	NR	A/BT	5.60±2.25/5.81±2.23	S	1 M	GCS
Yu 2012	30/30	33.38±12.65	A/BT	5.63±1.69/5.80±1.79	S	1 M	GCS
Feng 2005	44(29/15)/40(29/15)	39±15/36±13	A/BT	(3-5/9,6-8/35)/(3-5/3,6-8/32)	S	1 M	GCS/GOS
Liu 2015	43/42	NR	A/BT	NR	S	1 M	GOS
Liu 2010	15(9/6)/14(9/6)	32.6±15.3/31.9±15.8	EA/BT	(5/3,6/4,7/3,8/5)/(5/3,6/3,7/4,8/4)	S	U	GOS
Liu 2016	43(30/12)/42(29/14)	38.61±4.65/36.92±4.87	A/BT	3-5/22,6-8/35,9-11/17,12-15/11	S/Mo/Mi	30 D	GOS
Liu 2006	50(34/16)/50(32/18)	NR	EA/BT	(3-5/12,6-8/38)/(3-5/11,6-8/39)	S	30 D	GOS
Liu et.al. 2006	60(44/16)/60(42/18)	32.5/32	A+HO+LII/BT	(8/19,7/36,≤6/5)/(8/18,7/36,≤6/6)	S	30 D	GCS
Lu 2015	37(22/15)/37(22/15)	39.6±1.5/30.21±1.8	EA/BT	NR	S	30 D	GOS
Gu 2010	61(42/19)/61(41/20)	45±12.11/43.1±11.12	A+TCM/BT	(3-5/23,6-8/38)/(3-5/24,6-8/37)	S	1 W	GOS
Lv 2013	43(23/20)/42(25/17)	42.5±12.7/41.7±13.2	EA/BT	3.9±1.1/4.01±1.06	S	30 D	GCS/GOS
Tang 2016	15(11/4)/15(11/3)	49.53±15.73/52.14±11.36	EA/BT	5.80±1.47/5.57±1.22	S	4 W	GCS/GOS
Lou 2013	25/25	NR	A+HO/BT	NR	S	U	GOS
Sun 2009	30(22/8)/30(20/10)	45.2/39.8	A/BT	(3-5/9,6-8/21)/(3-5/8,6-8/22)	S	2 M	GOS
Song 2007	30/20	NR	EA/BT	NR	S	1 M	GOS
Zhang 2013	30(19/11)/30(21/9)	37.1±9.1/37.2±10.1	EA/BT	(3-5/13,6-8/17)/(3-5/12,6-8/18)	S	1 M	GOS
Zhang 2011	22(19/3)/20(14/6)	43.50±1.27/46.25±1.31	A/BT	6.41±1.47/6.45±1.47	S	30 D	GCS
Peng 2010	29(20/9)/27(20/7)	39.1±12.2/40.2±9.8	EA/BT	5.08±1.47/5.10±2.11	S	1 M	GOS
Cheng 2016	28/29	NR	EA/BT	5.42±1.29/5.68±1.54	S	U	GCS
Li 2003	40(29/11)/40(27/13)	39±15/36±14	A+TCM/BT	(3-5/9,6-8/31)/(3-5/8,6-8/32)	S	1 M	GOS
Li 2010	16(12/4)/14(10/4)	45.6/46.4	A+TCM/BT	(3-5/4,6-8/12)/(3-5/4,6-8/10)	S	1 M	GOS
Yang 2011	15(11/4)/15(10/5)	37.4/35.1	A+HO/BT	5.7/5.9	S	30 D	GCS
Yang 2007	20(11/9)/20(12/8)	40.25±0.36/42.06±0.28	EA/BT	6.5±1.471/6.6±1.04	S	7 D	GCS
Yang 2016	50/40	NR	A/BT	NR	S	60 D	GOS
Jiang 2009a	33(25/8)/30(26/4)	NR	A+HO/BT	NR	S	U	GOS
jiang 2009b	76(53/12)/58(48/10)	28.1/27.4	A/BT	5.3±1.18/5.1±1.21	S	U	GOS
You 2013	16(12/4)/15(10/5)	34.7±9.9/35.2±8.7	A/BT	6.2±1.7/5.9±1.4	S	4 W	GCS
Wang 2012	50(42/8)/50(40/10)	NR	A/BT	4.04±1.06/4.08±1.02	NR	1 M	GCS
Wang 2013	50/50	NR	EA+HO+TCM/BT	NR	S	2 W	GOS
Wang 2016	50(37/13)/50(37/13)	42.36±16.11/43.10±15.33	A/BT	5.90±1.53/5.98±1.60	S	3 W	GCS
Ge 2014	23(13/10)/23(15/8)	32.06±3.18/31.26±4.25	EA/BT	NR	S	1 M	GOS
Xie 2016	32(24/8)/32(25/7)	39.5/36.9	A/BT	4.92±1.17/4.81±1.55	S	1 M	GCS
Guo 2016	30(18/12)/30(20/10)	35.2±8.43/33.7±7.56	EA/BT	6.53±1.18/6.87±7.56	S	30 D	GCS
Chen 2013	39(25/14)/38(26/12)	31.47±3.88/31.52±3.82	A+HO/BT	NR	S	U	GOS
Chen 2011	36/34	NR	A+HO/BT	NR	S	1 M	GOS
Chen 2006	54(36/18)/51(37/14)	34±11/36±112	A+TCM/BT	(3-5/15,6-8/39)/(3-5/14,6-8/37)	S	8 W	GCS/GOS
Chen 2009	46(38/8)/46(35/11)	47±2.13/47±2.37	EA/BT	4.03±1.05/4.07±1.02	S/Mo	U	GCS
Lu 2014	20(12/8)/20(11/9)	34±116/33±16	A/BT	(5/6,6/7,7/3,8/4)/(5/5,6/6,7/4,8/5)	S	30 D	GOS
Tu 2010	51(35/16)/51(37/14)	42.5±11.4/41.1±11.2	A/BT	(3-5/16,6-8/35)/(3-5/17,6-8/34)	S	30 D	GOS
Yan 2018	50(32/18)/50(30/20)	35.2±11.8/34.5±10.3	EA/BT	4.6±1.15/4.5±1.44	S	1 M	GCS
Xue 2017	36(25/13)/38(24/12)	38.15±5.26/39.75±6.18	A+TCM/BT	5.75±2.26/6.13±2.27	S/Mo	8 W	GCS/GOS
Xu 2015	30(19/12)/30(18/11)	39.75±17.86/9.06±2.03	A/BT	6.0±1.80/5.9±1.70	S	14 D	GCS/GOS
Xing 2007	20/20	NR	A/BT	5.9±0.85/5.00±1.17	S	4 W	GCS
Wang et al 2016	39(22/17)/39(21/18)	53.5±2.4/53.4±2.5	A+HO/BT	6.3±1.36/6.4±1.14	NR	3 M	GCS
Ding 2015	40(24/16)/40(22/18)	35.8±4.3/35.4±4.3	A+TCM/BT	5.7±1.06/10.05±1.3	NR	U	GCS
Zhang 2014	80(82/44)/46	27.7	A+TCM/BT	NR	S	2 M	GOS
Tian 2012	58(85/31)/58	43.5	A/BT	8.5±3.01/8.46±3.22	S/Mo	14 D	GCS/GOS

NR: not report; A: acupuncture; EA: electroacupuncture; TCM: traditional Chinese medicine; HO: hyperbaric oxygen; BT: basic treatment; Mi: mild TBI; Mo: moderate TBI; S: severe TBI; M: month; W: week; D: day; U: unclear; GCS: Glasgow Coma Scale; GOS: Glasgow Outcome Scale.

**Table 2:** Results of subgroup analysis of GCS by treatment duration, TBI's degree, and intervention.

Variables		Numbers of studies	Numbers of patient(T/C)	MD(95%CI)	Heterogeneity(I2)	Z-value	*P*-value
Treatment duration	7d	1	20/20	2.20 [1.28, 3.12]	Not applicable	4.71	*P* < 0.00001
	14d	2	89/87	2.42 [1.56, 3.29]	0%	5.48	*P* < 0.00001
	3W	1	50/50	2.42 [1.29, 3.55]	Not applicable	4.18	*P* < 0.00001
	4W or 30d or 1M	12	358/354	2.20 [1.80, 2.59]	2%	10.9	*P* < 0.00001
	8W	2	91/91	2.13 [0.93, 3.33]	67%	3.47	*P* < 0.00001
	3M	1	39/39	3.20 [2.31, 4.09]	Not applicable	7.07	*P* < 0.00001
	Unclear	3	144/127	1.34 [-0.24, 2.92]	96%	2.02	*P*=0.04
	Test for subgroup differences				85.8%		*P* < 0.00001
TBI's Degree	Severe	17	560/548	2.00 [1.49, 2.51]	68%	7.69	*P* < 0.00001
	Severe/Moderate	2	91/91	2.13 [0.93, 3.33]	67%	3.47	*P* = 0.0005
	Unclear	3	129/129	2.03 [-0.06, 4.12]	95%	1.91	*P*=0.06
	Test for subgroup differences				0%		P = 0.98
Intervention	A	12	425/415	2.22 [1.47, 2.96]	83%	6.07	*P* < 0.00001
	EA	8	248/248	2.13 [1.59, 2.68]	29%	7.71	*P* < 0.00001
	A plus TCM	2	85/85	0.83 [-0.49, 2.15]	84%	1.23	*P* = 0.22
	Test for subgroup differences				43.4%		*P* = 0.17
Total		22	780/768	2.03 [1.54, 2.52]	78%	8.17	*P* < 0.00001

## Data Availability

The data supporting this systematic review are from previous studies and datasets, which have been cited. The processed data are available from the corresponding author upon request.
